# Sulfide silver autometallography to differentiate the ultrastructural localization of iron-carbohydrate complexes inside macrophages

**DOI:** 10.1038/s41598-025-27257-6

**Published:** 2025-12-08

**Authors:** Anne-Marinette Cao, Jules Duruz, Mauro Sousa de Almeida, Dimitri Vanhecke, Amy E. Alston, Reinaldo Digigow, Beat Flühmann, Alke Petri-Fink, Barbara Rothen-Rutishauser

**Affiliations:** 1 Adolphe Merkle Institute, University of Fribourg, Chemin des Verdiers 4, Fribourg, Switzerland; 2 CSL Vifor, Glattbrugg, Switzerland; 3 Present Address: National Center of Competence in Research Bio-Inspired Materials, University of Fribourg, Chemin des Verdiers 4, Fribourg, Switzerland

**Keywords:** Iron sucrose, Ferric carboxymaltose, Sulfide silver autometallography, Electron microscopy, Biodistribution, Drug delivery, Iron, Transmission electron microscopy, Preclinical research

## Abstract

Iron is an essential element for numerous physiological processes in the human body, and maintaining proper iron homeostasis is critical for health. Iron imbalance can lead to conditions like iron deficiency anemia (IDA). Intravenous (IV) iron drugs, including iron-carbohydrate complexes such as iron sucrose (IS) and ferric carboxymaltose (FCM), are commonly used in the treatment of IDA. However, the cellular mechanisms underlying the uptake and intracellular fate of these complexes remain poorly understood despite over seven decades of clinical use. This study introduces a novel application of sulfide silver autometallography (ssAMG) to track iron inside macrophages treated with two widely used intravenous iron products, IS and FCM. Using this technique, the ultrastructural localization of iron in macrophages was visualized in comparison to ferric ion (Fe^3+^). ssAMG improved the visualization of IS and FCM, revealed as crystalline, cluster-like particles, i.e. silver precipitates, localized inside intracellular vesicles. Interestingly, for both iron-carbohydrate complexes, the silver precipitates were observed exclusively inside the cells and not on the cell surface as seen for ferric ion. These results suggest that IS and FCM are not internalized by macrophages as ferric iron bound to transferrin and provide new insights into the cellular processing of iron-carbohydrate complexes to advance the understanding of iron homeostasis.

**Supplementary Information:**

The online version contains supplementary material available at 10.1038/s41598-025-27257-6.

## Introduction

Iron is crucial for various processes in the human body, such as oxygen transport, DNA synthesis, enzyme function, immune system, and antioxidant defense^1^. It is thus vital that adequate concentrations of iron are maintained accessible and at sufficient concentration for physiological needs at both cellular and systemic levels. Because iron is toxic in excess, iron must be homeostatically regulated and stored to prevent cellular damage and overload-induced apoptosis, known as ferroptosis. In physiological conditions, iron homeostasis is meticulously balanced through a combination of highly regulated iron absorption, transport, storage, utilization, and regulation^2,3^.

The physiology of iron metabolism has been elucidated over the last decades. It begins with iron absorption, generally from the diet. Ferric iron (Fe^3+^) is the most common form of dietary iron, but the soluble form that can be taken up by cells is ferrous iron (Fe^2+^). In the gastrointestinal tract, ferric iron is thus reduced to ferrous iron by a membrane-bound reductase named duodenal cytochrome B (DCYTB), which is expressed on the apical brush border membrane of intestinal epithelial cells^4^. Ferrous iron is then transported by the divalent metal-ion transporter 1 (DMT1) to cross the apical membrane of enterocytes, and subsequently exported by ferroportin 1 to cross the basolateral membrane of enterocytes to enter the systemic circulation bound to serum transferrin. Once absorbed, ferrous iron is oxidized back to ferric iron by ferroxidases such as caeruloplasmin and hephaestin^2,5^.

In addition to the uptake of iron by dietary intake, red blood cell recycling orchestrated by liver and spleen macrophages is also an important part of systemic iron homeostasis^6^. The primary transporter of ferric iron in the plasma is transferrin. This is a single-chain glycoprotein displaying a bi-lobal structure, with each lobe exhibiting high-affinity yet reversible and pH-sensitive binding to Fe^3+^. Therefore, transferrin can bind to two Fe^3+^ and does not bind effectively to Fe^2+^^3^. Transferrin-bound iron (TBI), upon delivery, is recognized by and bound to transferrin receptors on cells in target tissues^7^. The TBI-transferrin-receptor complex then undergoes endocytosis, allowing Fe^3+^ to be released from transferrin by the decrease in pH inside lysosomes, next reduced by the reductase six-transmembrane epithelial antigen of the prostate 3 (STEAP3) into Fe^2+^. Ferrous iron can then be transported across the endosomal membrane via DMT1 and released in the cytoplasm, where it can be stored, utilized, and exported throughout the cell^5,8–10^. When transferrin is saturated with Fe^3+^ and serum iron levels are high, as in iron overload diseases such as hemochromatosis, non-transferrin-bound iron (NTBI) can also be present in plasma. NTBI includes plasma iron forms not bound to transferrin, ferritin, or heme. Many cell types can take up NTBI, yet its mechanism is less well understood due to the heterogeneous and transient nature of NTBI^11^.

Iron deficiency anemia (IDA) is the most common form of anemia and the most common nutritional deficiency worldwide. IDA affects approximately 30% of the human population across age groups^12–15^. Oral and intravenous (IV) iron are both options for the treatment of IDA. When oral iron salts are not well-tolerated or there is a need for a more rapid iron repletion, IV iron-carbohydrate complexes are often prescribed. The clinically available iron-carbohydrate complexes are nanoparticles comprised of a polynuclear ferric iron core with bound carbohydrate ligands of various structures that promote colloidal stability. Two commonly used products are iron sucrose (IS) and ferric carboxymaltose (FCM), which have different physicochemical properties, pharmacokinetic profiles and dosing strategies^16^. The presumed biodisposition of the iron carbohydrate complexes after administration is clearance from plasma via cellular uptake. This is hypothesized to be predominantly orchestrated by liver and spleen macrophages^17^ and the complexes are biodegraded to liberate bioavailable iron via normal iron homeostatic mechanisms^16^.

Despite widespread clinical use over many decades, the understanding of the mechanism of action at the cellular level, including cellular uptake, intracellular fate, and metabolism, of these iron-carbohydrate complexes remains limited. Investigating this aspect of the mechanism of action of these complexes is critical in understanding the different pharmacokinetic profiles and the impact of disease on bioavailability^18^. Indeed, tracking the cellular fate of iron released from IV iron-carbohydrate complexes has been challenging because it is often prohibitively difficult methodologically to differentiate exogenous iron from endogenous iron. First, both are trafficked intracellularly similarly by iron-binding proteins like transferrin and ferritin. Second, iron is constantly recycled and dynamically exchanged between storage and functional pools. Additionally, colorimetric methods, such as Prussian blue, or fluorescent techniques often limit the detection of iron intracellularly due to accumulation on the membrane^19^.

To circumvent these challenges and enable further exploration of intracellular handling of iron-carbohydrate complexes, we reinvented sulfide silver autometallography (ssAMG), making use of heavy metals that are compatible with electron microscopy and coupling with specific staining to render iron visualizable at the ultrastructural level. Autometallography (AMG) is widely used in tissue section staining for the revelation of heavy metals such as gold, silver, zinc and mercury^20,21^. In principle, metals or metals with sulfur and/or selenium containing AMG nanocrystals that can be silver-amplified are exposed to a protective colloid to form microscopically detectable silver precipitates^22^. The silver precipitates report the localization of AMG nanocrystal sources in biological samples and, thus, indicate the localization of iron. ssAMG was optimized and applied as a new tool to visualize the ultrastructural localization of iron inside cells. Phagocytic immune cells are among the first cells exposed to administered iron, and the murine macrophage J774.1 cells were used in this study^23^. Together, this method thus allowed us to detect iron and monitor the fate of iron in macrophages treated with iron-carbohydrate complexes (IS and FCM).

## Results

### Sulfide silver autometallography presents an alternative to the Prussian blue assay for detecting iron in cells

**Fig. 1:**
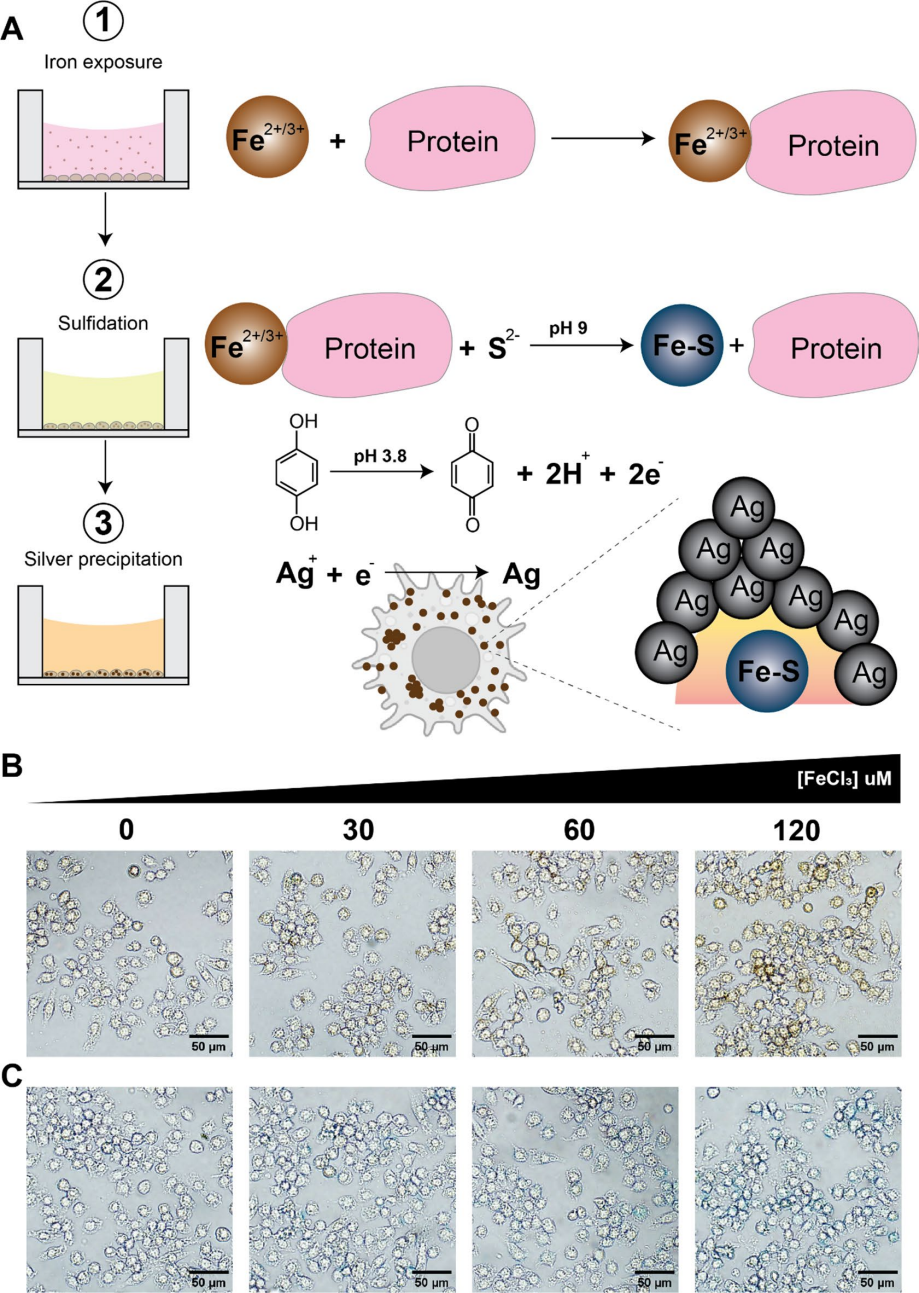
Detection of iron in macrophages using sulfide silver autometallography. (**A**) Scheme of workflow including three main steps: iron exposure, in which the cells (J774A.1) are subjected to a treatment with iron solution; sulfidation, in which the cells are incubated in a sulfur solution allowing iron sulfide (Fe–S) to form; and silver precipitation, in which silver atoms (Ag) reduced from silver ions (Ag+) accumulate on Fe–S AMG nanocrystals. Gradient staining of J774A.1 cells treated with increasing concentrations of iron using ssAMG (**B**) and Prussian blue (**C**).

An experimental approach was designed to study iron uptake and distribution in macrophages (Fig. 1A). J774.1 murine macrophages were used as a representative cell type relevant for iron uptake in the bloodstream upon iron administration. First, the cells were exposed to different forms of iron, i.e. iron (III) chloride and iron-carbohydrate complexes (IS and FCM), and iron-protein complexes were allowed to form. Second, the iron-exposed and fixed cells underwent a sulfidation where iron (II and III) sulfides, represented as Fe–S, were formed; AMG nanocrystals were created; and associated proteins to the iron core were released. Third, the AMG process occurred in the presence of hydroquinone and a protective colloid (gum arabic), where silver ions (Ag+) were reduced to silver atoms (Ag) and deposited on catalytic clusters triggered by Fe–S AMG nanocrystals.

A physiological case was built to study ferric iron (Fe^3+^). J774A.1 macrophages were exposed to increasing concentrations of FeCl_3_ ranging from 30 to 120 µM for 6 h and subsequently performed ssAMG. A gradient of increasing dark yellow staining inside macrophages was imaged using optical light microscopy (Fig. 1B). In parallel to this experiment, Perl’s Prussian blue staining was performed on J774A.1 cells treated with the same FeCl_3_ concentrations: the typical blue gradient demonstrated the presence of iron was observed as expected (Fig. 1C) and was perfectly comparable to the dark yellow gradient obtained by the optimized ssAMG in Fig. 1B. Moreover, the cell morphology did not change in the presence of all ferric ion concentrations and the cells looked viable. Noteworthy, the experiment performed at 120 µM ferric iron showed the strongest staining among four tested concentrations, indicating iron detection inside macrophages, at a concentration 15 times lower than the commonly studied, clinically relevant concentration of iron-carbohydrate complexes (1800 µM)^24^. Therefore, the optimized ssAMG not only enabled the detection of iron as sensitive as the standard method, i.e. Perl’s Prussian blue staining, but also provided a promising alternative to investigate iron-carbohydrate complexes.

### Significantly improved visualization and ultrastructural localization of iron prove the cellular uptake and distribution of ferric iron

**Fig. 2:**
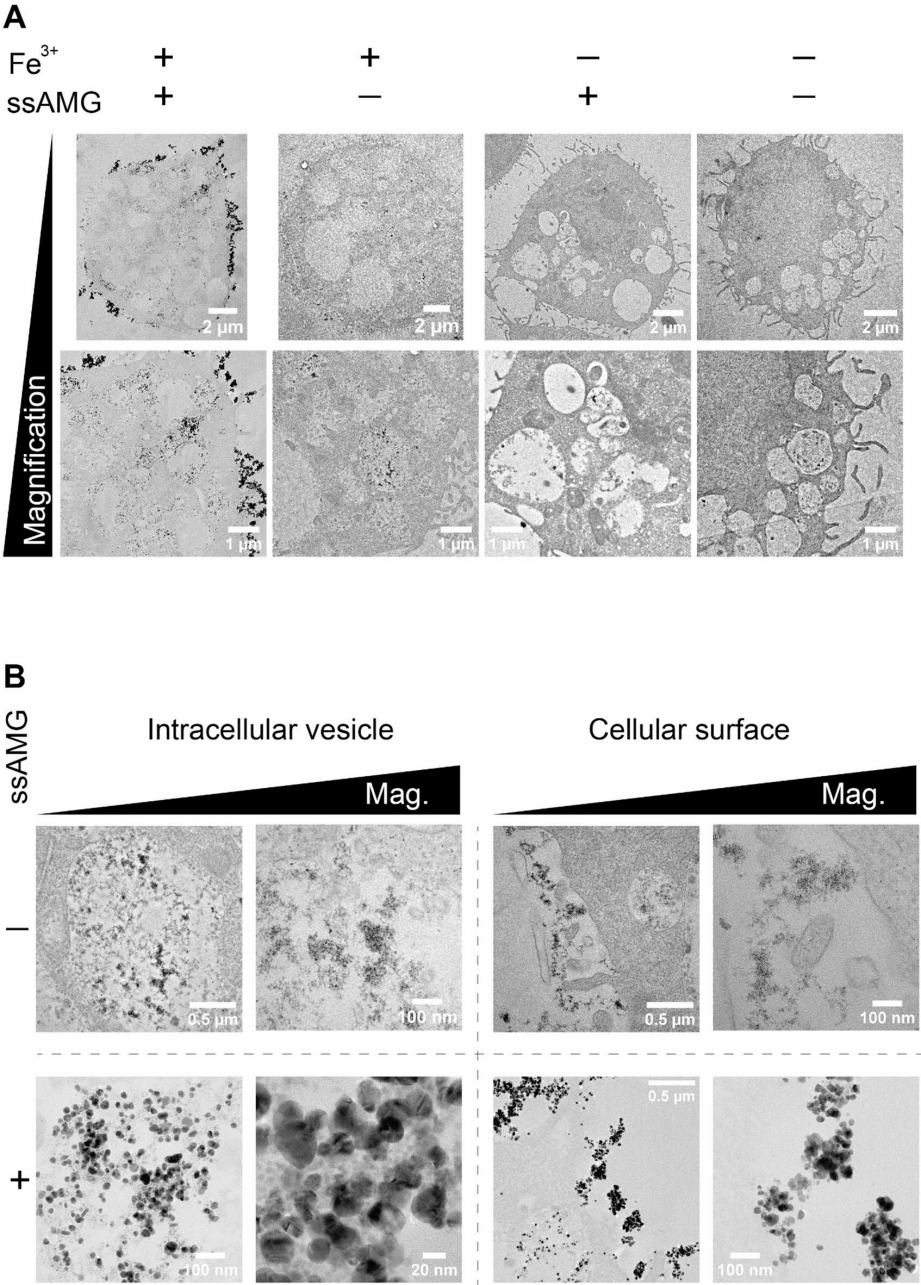
Visualization and ultrastructural localization of ferric iron in macrophages. (A) Macrophages treated with ferric iron and imaged using ssAMG (first column on the left) showed high-contrast silver precipitates (black clusters), whereas the three other controls did not. Representative whole-cell images are shown on the top row, and each corresponding zoom-in is in the bottom row. (B) ssAMG significantly improves the visualization of iron and shows two ultrastructural localizations of iron inside cellular vesicles or at the cellular surface (bottom row (+)). “Mag.” stands for “Magnification”.

Following up on the clear proof of iron detection by ssAMG using light microscopy, the approach shown in Fig. 1A was then expanded to study the localization of iron in macrophages at the ultrastructural level. Ferric iron (Fe^3+^) was used, and it was hypothesized that, according to the physiological iron homeostasis, two main localizations of Fe^3+^ interacting with macrophages could be observed. First, ferric iron bound to plasma transferrin could be taken up by J774A.1 cells through transferrin receptors^23^, or via other receptor-mediated uptake (e.g. clathrin) mechanisms. The precise mechanisms of iron carbohydrate complex uptake have not been well studied to date. Second, internalized iron, either delivered to and/or stored in cellular compartments such as the endolysosomal system, was expected to be observed inside macrophages and inside vesicular compartments. J774A.1 cells were exposed to 120 µM of ferric iron (Fe^3+^) for 24 h, followed by ssAMG and TEM sample preparation (See Materials and Methods). Control experiments included iron-exposed cells but no ssAMG (unlabeled control), ssAMG but no iron exposure (false positive control), and neither iron nor ssAMG (false negative control). As a result, silver precipitates, observed as black clusters and seen exclusively with macrophages treated with Fe^3+^ followed by ssAMG, reported a clear detection of iron at the ultrastructural level. On the contrary, in all three control experiments, the ultrastructure of the cells was well-defined, but iron could not be visualized (Fig. 2A).

Focusing on macrophages receiving Fe^3+^ treatment, two main localizations of silver precipitates were found: at the outer cell membrane of macrophages and in intracellular vesicles. Ultramicrographs at sub-nanometer resolution revealed cluster-like patterns of silver precipitates, illustrating the accumulation of silver atoms during ssAMG. This observation also corresponded to the expected localizations of Fe^3+^ (Fig. 2B). The high density, crystal induced-Bragg reflections and rounded-off shape of the precipitates stood out clearly against the cellular ultrastructure and was easily recognized – there was no confusion with other cellular structures (Fig. 2B, bottom row).

In addition, events of higher density were observed in the unlabeled control, i.e., iron-exposed but not ssAMG-treated samples. The interpretation of these events remains inconclusive as there was no information available that the increased density was indeed caused by iron. However, unlike the ssAMG-induced precipitates, these events were clearly not crystalline (Fig. 2B, top row).

Altogether, the results showed a great improvement in the visibility and recognizability of intracellular iron, promising a useful application of ssAMG in studying the uptake and distribution of iron-carbohydrate complexes.

### Sulfide silver autometallography reveals ultrastructural localizations of iron-carbohydrate complexes internalized into macrophages

Since the optimized ssAMG succeeded in elucidating the fate of Fe^3+^ inside macrophages, we moved on to apply the experimental method shown in (Fig. 1A) to study IS and FCM, two effective IV iron formulations commonly used in clinics, but whose uptake and intracellular distribution remain unclear. It has been hypothesized that iron-carbohydrate complexes undergo uptake via two potential mechanisms. The majority of intact complexes may be associated with plasma proteins and can be taken up by macrophages and degraded to release iron. A much smaller fraction of iron is released from the iron complexes in plasma which binds directly to plasma transferrin. The proportion of direct iron release is related to the stability of the iron-carbohydrate complexes. If transferrin becomes oversaturated, there may be availability of labile iron to participate in redox reactions^18^. We performed 24-hour exposure of iron-carbohydrate complexes (IS and FCM) at increasing concentrations from **0.1** mg/mL to **1** mg/mL to J774A.1 macrophage, followed by ssAMG and Prussian blue and imaged by light microscopy. The increase in IS dosage resulted in an increasing staining gradient but severely affected cell density and morphology at the highest concentration (Fig. S1), whereas for FCM, it was unclear to detect iron at the whole-cell level with light microscopy (Fig. S2). The concentrations (**0.1**-**1** mg/ml) corresponding to the limits of reasonable physiological exposure of total iron for single doses (50–500 mg)^24,25^ were chosen for further investigation. Osmium fixation and ultrathin section preparation were then performed, and macrophages treated with **0.1** and **1** mg/ml IS and FCM were imaged by TEM as described in the Materials and Methods section.

**Fig. 3:**
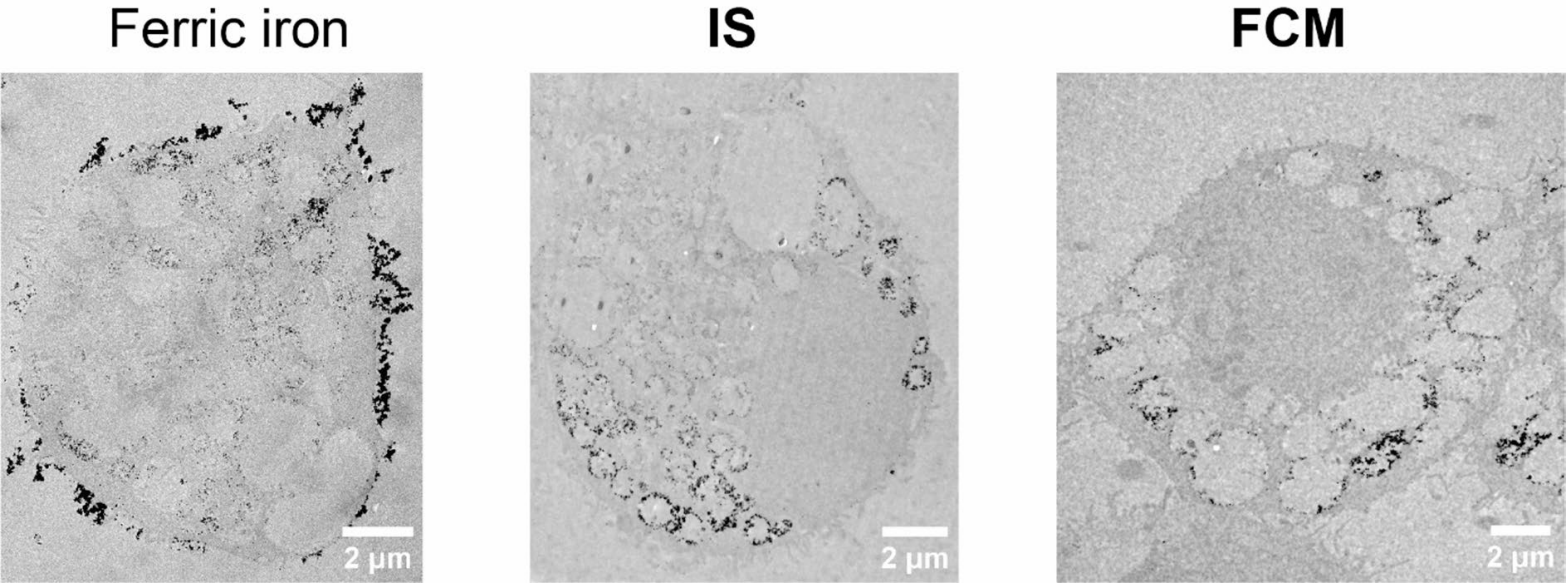
Representative whole-cell images of macrophages taken iron up from different sources. (*Left to right*) Representative images of macrophages treated with 120 µM of ferric iron (Fe^3+^), lower dose of IS (0.1 mg/ml), and higher dose of FCM (1 mg/ml).

It was immediately recognizable that, at the single-cell level, silver precipitates localized exclusively inside macrophages treated with IS and FCM but not at the outer cell membrane, differing them from Fe^3+^-exposed cells (Fig. 3). This result demonstrated that either the iron-carbohydrate complexes did not degrade into iron ions (that could be bound to transferrin), even did not degrade at all, or resulted in undetectable amount of iron ions. This also suggested that IS and FCM remained intact complexes while being taken up by the cells. Once they reached digestion and storage compartments inside the cells, the iron-carbohydrate complexes might have undergone metabolic treatment that resulted in iron-protein complexes which could react with sulfur to form iron sulfides to initiate ssAMG, forming silver precipitates inside the cells.

**Fig. 4:**
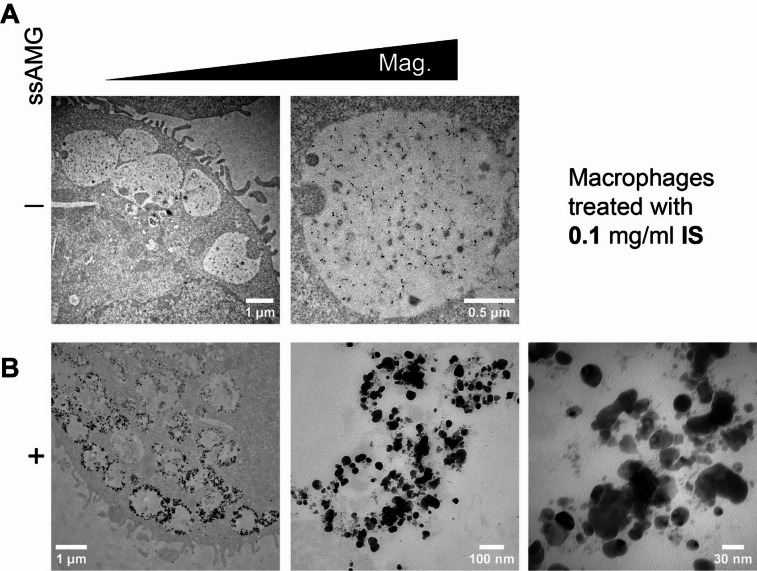
Visualization and ultrastructural localization of IS. Compared to the control of macrophages treated with the lower dose (0.1 mg/ml) of IS (**A**), the use of ssAMG (**B**) clearly revealed the presence of iron indicated by high-contrast silver precipitates (black clusters) at the subcellular level. “Mag.” stands for “Magnification”.

**Fig. 5:**
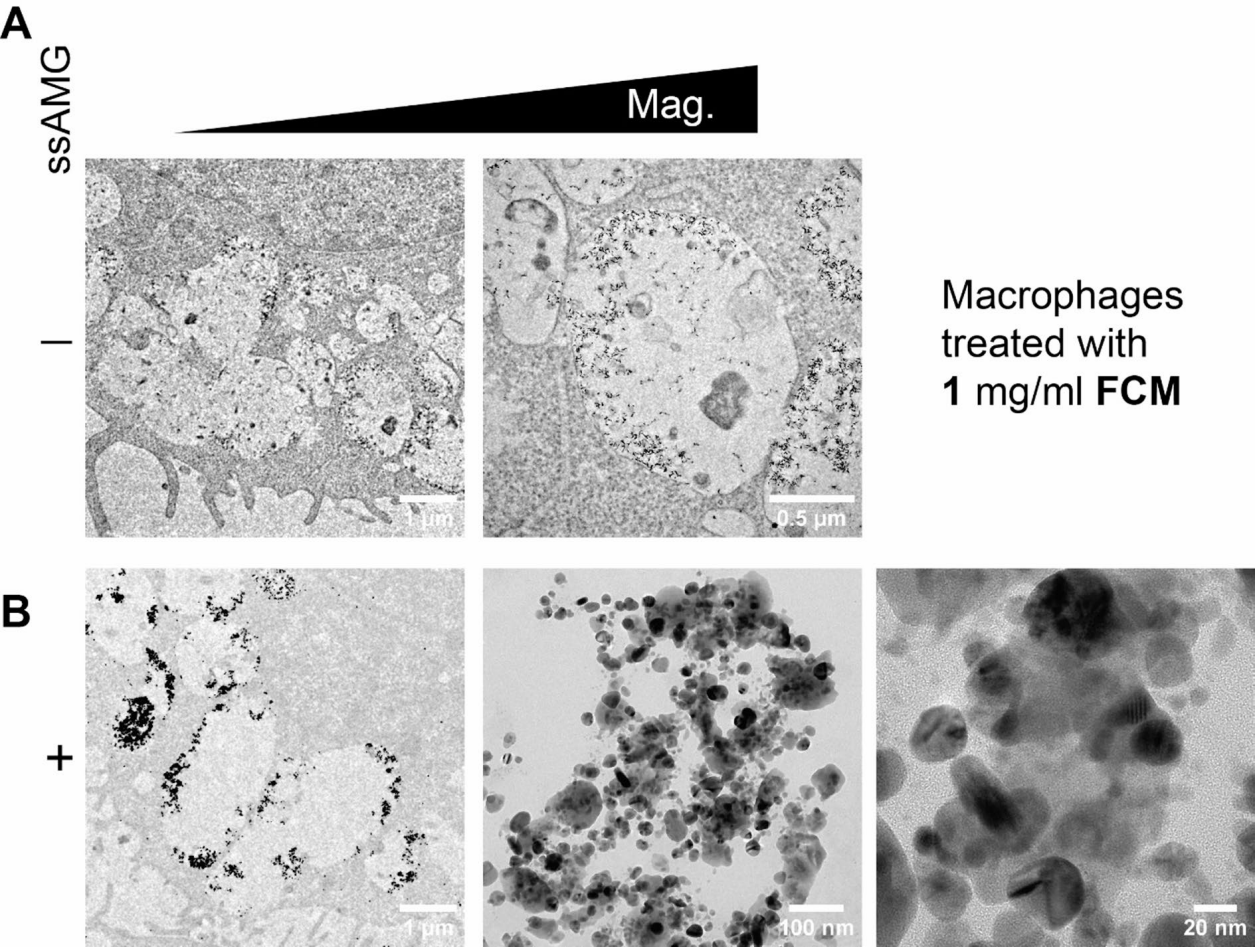
Visualization and ultrastructural localization of FCM. Compared to the control of macrophages treated with the higher dose (1 mg/ml) of FCM (**A**), the use of ssAMG (**B**) clearly revealed the presence of iron indicated by high-contrast silver precipitates (black clusters) at the subcellular level. “Mag.” stands for “Magnification”.

Iron-exposed cells treated with and without ssAMG (namely “unlabeled control”) were imaged at the ultrastructural level in a TEM (Figs. 4 and 5). Despite high concentrations of iron exposed to the cells, iron cannot be observed without the ssAMG treatment (Fig. 4A for the treatments with IS and Fig. 5A for FCM). On the contrary, ssAMG greatly improved the recognizability of iron, revealed as crystalline, cluster-like particles, i.e., silver precipitates, localized inside intracellular vesicles (Figs. 4B and 5B). Interestingly, this was true for both IS and FCM: silver precipitates were observed exclusively inside the cells and not on the cell surface. Finally, because of the striking structural appearance of iron after ssAMG treatment in the TEM, the distribution of iron uncovered the intracellular presence of iron-carbohydrate complexes. This finding provided, for the first time, insight into the fate of iron-carbohydrate complexes after cellular internalization.

Of note, a “concentration effect” was consistently found to differ while comparing IS and FCM. At **0.1** mg/ml, IS showed a clear intracellular biodistribution (Fig. 4) whereas FCM internalization was only observed at the ultrastructural level (Fig. S3). At **1** mg/ml, FCM did not interfere with cell proliferation but IS showed a severe impact on the cell density and morphology (Fig. S1, S4).

## Discussion

In this study, we repurposed an autometallography (AMG) method to harness its remarkable sensitivity for detecting heavy metals, specifically by employing iron sulfide as a catalytic agent^22^. Unlike traditional AMG approaches that aim to precipitate iron directly, our method leverages iron sulfide to initiate the nucleation of nanocrystals, ultimately leading to the accumulation of silver atoms and the formation of characteristic “silver precipitates.” These precipitates are readily detectable by both light and electron microscopy^21^. We optimized the protocol to enable high-resolution ultrastructural localization of iron within macrophages, providing, for the first time, direct evidence of the cellular internalization of iron carbohydrate complexes, including IS and FCM. As described previously, advancing understanding of the biodisposition of iron-carbohydrate nanomedicines has been hindered by the lack of *in vivo* bioassays that can measure nanoparticle-derived iron and distinguish this concentration from endogenous iron^18^. Data that can advance the understanding of the rate and extent biodegradation of iron-carbohydrate complexes intracellularly is urgently needed to provide a mechanistic rationale for the variable plasma pharmacokinetic profiles observed *in vivo*^26^. Moreover, addressing this research gap can facilitate understanding of how diseases commonly treated with iron-carbohydrate complexes affect the biodisposition, especially highly prevalent diseases associated with concomitant inflammatory burdens (e.g. chronic kidney disease and heart failure)^27^.

Our ssAMG technique demonstrated a remarkable degree of sensitivity, capable of detecting iron concentrations as low as 100 femtogram per cell. This exceptional sensitivity also means that ssAMG is vulnerable to impurities, which can potentially induce artifacts, a well-documented phenomenon in high-sensitivity methodologies^28,29^. Despite this high sensitivity, ssAMG is an inherently chemistry-based method. The chemistry of endogenous and exogenous Fe^3+^ is the same; therefore, ssAMG cannot differentiate between these two pools. The morphology of the crystalline silver precipitates, exhibiting diffraction contrast on top of a significantly higher electron density compared to any other element within a biological context, ensures the reliability of the detection. The likelihood of false positives, e.g. confusion between sporadic dark stain artifacts and Ag crystals, is minimal. For the same reason, the probability of false negatives is also low. Taken together, all prerequisites for implementing Fe^+ 3^ quantification routines are in place. Nevertheless, our study was conceived for qualitative analysis, where sampling and technical requirements are not optimal for accurate quantification^30–32^ but are perfectly satisfying for the focus on iron detection in relevant biological applications, i.e. dietary iron and uptake of iron carbohydrate complexes.

A physiological case was built where macrophages were exposed to an increasing dose of ferric iron (Fe^3+^), representing the most common source of dietary iron. Iron exposure was maintained for 24 h, allowing balanced iron homeostasis to be achieved, prior to a parallel comparison of iron detection using ssAMG and Prussian blue. This results in brownish staining that is clearly visible, and more conclusive than the Prussian blue staining. Whereas Prussian blue is sensitive to ferric iron, in ssAMG, both ferrous and ferric states contribute to the formation of iron sulfide (noted as Fe-S), which serves as the catalyst of silver precipitation. Therefore, there is no ferrous ↔ ferric conversion needed in ssAMG. This also suggests that ssAMG can be further optimized for a quantitative process, for example, to directly determine the total iron content. The technical simplicity yet impressive sensitivity of ssAMG, combined with its compatibility with TEM, make this method uniquely advantageous in the detection of iron. Indeed, it is worth pointing out the exclusiveness of iron-exposed and ssAMG-treated cells in affirming iron detection over the other three controls (Fig. 2A). This finding substantiates the enhanced sensitivity of the ssAMG method, attributable to the discernible separation of iron-induced silver precipitates from the endogenous iron present in the cellular background.

We chose to explore the ultrastructural localization with a focus on the cellular uptake mechanism. Silver precipitates were present at the cellular surface and in intracellular vesicles of J774A.1 macrophage. When present inside macrophages, these precipitates localize exclusively in intracellular vesicles. The conditions we employed for both ssAMG (sulfidation at high basicity followed by silver precipitation at high acidity) and TEM sampling (OsO_4_ fixation, dehydration, polymerization, and ultrathin sectioning) were notably non-physiologic, making ssAMG incompatible with co-staining of fluorescent dyes to determine specific intracellular organelles, which often need to be performed with unfixed cells^33^. The experimental design was formulated with a minimal iron concentration (0.1 pg/cell), making iron overload unlikely. Hence, the finding is well in agreement with the known uptake of ferric iron by transferrin and transferrin receptors, which are expressed by macrophages^23^. Macrophages were chosen as a model not only because of their relevance in iron homeostasis^34^, but also because multiple pathways potentially involving both NTBI and intact complexes have been proposed for the uptake of iron-carbohydrate complexes^26^. The experimental models using macrophages thus satisfy physiological relevance and technical demands to study the fate of iron in cells. Other approaches, such as fluorescent probes for iron^19^, have been developed. Although the method using ssAMG is not compatible with live-cell imaging, nor is it commercially available, it provides a way to specifically localize iron in a high-resolution cellular context.

The method was then applied to study cellular uptake and intracellular localization of iron-carbohydrate complexes in macrophages. It is unclear how iron-carbohydrate complexes, such as IS and FCM, are taken up and transported at the molecular level, and clear-cut evidence of cellular internalization of these iron products is still missing. It has been proposed that iron-carbohydrate complexes remain mainly intact, which promotes interaction with plasma proteins other than transferrin to facilitate uptake by macrophages. A small portion of iron-carbohydrate complexes has been suggested to be degraded into transient labile iron that would then be bound by plasma transferrin^18^. Our results showed that, in the used cell culture medium, both IS and FCM are internalized differently by macrophages, as expected from the physiological way of ferric iron through TBI. Indeed, in macrophages exposed to iron-carbohydrate complexes, silver precipitates were found exclusively in intracellular vesicles and were not seen at the outer cell membrane of macrophages, where transferrin receptors are located. This observation differs from the physiological case where ferric iron-induced macrophages showed both localizations of silver precipitates (at the outer cell membrane and in intracellular vesicles). This means that, for both iron-carbohydrate complexes, taken-up iron was not transferred as ferric iron bound to transferrin. Several explanations account for this result. First, the hypothesized portion of the degraded iron formulation must be either negligible or unbound to transferrin (and subsequently unbound to transferrin receptors). Second, IS and FCM used in this experiment remained highly intact in the used cell culture medium (i.e., complete RPMI). Third, the “concentration effect” consistently observed at the same concentration of IS and FCM but differently affecting macrophages in cell density and morphology (IS at **1** mg/ml) indicates different interaction of the two complexes at the cellular level. This observation is well aligned with the recent findings highlighting the fast-release kinetics but leading to transient toxicity of IS and the delayed processing via the “Hamster effect” of FCM^35^. Expanding this model to include human plasma exposure would improve the model’s bio-relevance and will contribute substantially to understanding the metabolic fate of the iron-carbohydrate complexes. The findings are well in agreement with quantitative proteomics data previously reported^24^ and bring complementary evidence supporting the internalization of intact iron-carbohydrate complexes into macrophages^18,35^.

Altogether, the optimized ssAMG approach is a tool that is highly sensitive for detecting iron derived from iron-carbohydrate complexes, which greatly improves its detection at the ultrastructural level and, importantly, provides precise spatial information. Thus, the use of ssAMG can provide crucial data to gain insights into the intracellular biodistribution of iron-carbohydrate complexes.

## Materials and methods

### Materials

Murine macrophage J774A.1 cells were obtained from American Type Culture Collection (ATCC^®^ TIB-67™). Culture medium Roswell Park Memorial Institute 1640 (RPMI-1640, 42401-018), phosphate-buffered saline (PBS), and supplementary reagents, including fetal bovine serum (FBS, 10270-106), L-glutamine (25030-024), and penicillin/streptomycin (15140-122), were purchased from Gibco (Life Technologies Europe B.V., Switzerland). Chemicals were purchased from Sigma-Aldrich: acetone (1.00014), sucrose (84100), ammonium sulfide (12135-76-1), silver lactate (15768-18-0), hydroquinone (123-31-9), gum arabic (9000-01-5), citric acid (77-92-9), osmium tetraoxide (251755), potassium ferrocyanide (455989), sodium citrate (6132-04-3), and epoxy embedding medium kit (45359). Hydrochloric acid was from VWR (20252.290), sodium cacodylate from Polysciences (18661-500), ethanol from Reactolab (64-17-5), and glutaraldehyde (111-30-8) from Electron Microscopy Sciences. Iron (III) chloride was purchased from Sigma-Aldrich (10025-77-1). IV iron carbohydrates, including iron sucrose (IS, 20 mg/mL) and ferric carboxymaltose (FCM, 50 mg/mL) were obtained from CSL Vifor (St. Gallen, Switzerland). Cultureware was from Ibidi (µ-slide 8-well, 80807), Nunc (4-well chamber slide, 154526PK) and Falcon (12-mm glass coverslip, 12-well and 24-well plates).

### Methods

#### Cell culture

J774A.1 cells were cultured in RPMI-1640 supplemented with 10% heat-inactivated FBS, 2 mM L-glutamine, 100 IU/ml penicillin, and 100 µg/ml streptomycin at 37 °C in humidified air with 5% CO_2_. At ~ 80% confluency, the cells were subjected to subculture or seeding performed by gently scraping followed by well resuspending in the completed culture medium at the desired concentrations. Empirically, J774A.1 cells were seeded at a cell density of ~ 10^5^ cells/cm^2^. Subculture was performed every 5–7 days, and seeded cells were maintained 24 h at the described conditions. When planned for ssAMG followed by sample preparation for transmission electron microscopy (TEM), cell seeding was performed on sterile glass coverslips inserted in a 12-well culture plate in order to be easily transferred to a clean plate where thorough washes were performed between critical steps (see Sect. *4.2.3*).

#### Iron exposure

Where applicable, J774A.1 cells were exposed to either a gradient of iron (III) chloride (FeCl_3_) ranging from 30, 60, to 120 µM or iron-carbohydrate complexes (IS and FCM) at 0.1 and 1 mg Fe/ml, i.e., 1.8 and 18 mM, Fe respectively. After exposure, the cells were carefully washed twice with pre-warmed PBS.

#### Sulfide silver autometallography (ssAMG)

The ssAMG process includes 3 steps: cell fixation, sulfidation, and silver precipitation. Samples can be imaged with both light and electron microscopies. For electron microscopy, it is essential that the samples must be thoroughly washed between the 3 steps. To this end, samples subjected to be imaged by electron microscopy were prepared on glass coverslips inserted in a 12-well plate (Falcon), and the washing included the transfer of the coverslips to new 12-well plates to eliminate left-over contaminates. For light microscopy, samples were prepared in either 8-well (Ibidi) or 4-well (Nunc) chamber slides where both the 3-step staining and washes were performed.

Solutions used for the precipitation step were prepared as follows:

- Protecting colloid: 20 g crystalline gum Arabic was dissolved in 100 ml Milli-Q water by intermittent stirring for 3 days. The solution was filtered through layers of gauze, aliquoted, and stored at -20 °C.
- Na-citrate buffer: 25.5 g citric acid and 23.5 g sodium citrate dihydrate were completely dissolved in Milli-Q water heated to 40 °C. The total volume was adjusted to 100 ml and the pH to 3.8. The buffer can be kept fresh for a year.
- Reducing agent: 0.85 g hydroquinone was completely dissolved in 15 ml Milli-Q water heated to 40 °C. The hydroquinone solution must be used immediately after being prepared, and the temperature of the solution must not at any time exceed 45 °C.
- Silver ion supply: 0.11 g silver lactate was dissolved in 15 ml Milli-Q water. The solution must be protected from light and can be aliquoted and stored at -20 °C.
- The developer mixture, containing 60% (v/v) gum arabic, 10% Na-citrate buffer, 15% hydroquinone, and 15% silver lactate, was prepared just before use.

First, cell fixation was performed with 2% glutaraldehyde in 0.1 M Na-cacodylate with 0.1 M sucrose (pH 7.2) for 2 h at room temperature, followed by 3 rinses with Milli-Q water. The glass coverslips covered with fixed cells were carefully transferred into a new 12-well plate containing 1 ml of Milli-Q water in each well.

Second, the sulfidation was performed with 1% (v/v) ammonium sulfide in EtOH 70% (pH 9) for 15 min at room temperature, immediately followed by 2 washes with Milli-Q water. The coverslips were then taken out by tweezers and quickly submerged in Milli-Q water before being transferred to a new 12-well plate as previously described. Three more extensive washes with Milli-Q water were performed.

Third, the silver precipitation was performed with the developer mixture added to each well, incubated for 20 min at 26 °C, and protected from light, e.g. completely covered by aluminum foil. Thorough washes were performed as described in the previous steps.

#### Perl’s Prussian blue staining

J774A.1 macrophages were seeded in 4-well chamber slides (Nunc) and exposed to different forms of iron. The cells were then fixed in glutaraldehyde, as previously described. A total volume of 500 µL of 3.3 M HCl and 5% Potassium ferrocyanide (1:1) was added to each well and incubated for 30 min at room temperature. Two washes with PBS were performed, and the chambers were removed, allowing to mount a coverslip on the slide with Fluoromount-G Mounting Medium. Images were acquired with an Olympus BX51 microscope equipped with an Olympus DP72 camera.

#### Transmission electron microscopy (TEM)

Samples were post-fixed in 1% OsO_4_ (Sigma-Aldrich, 251755) in Milli-Q water for one hour at room temperature. The OsO_4_ solution was applied directly on top of the cells in a 24-well cell culture plate. The samples were washed three times with Milli-Q water and subsequently dehydrated in a series of 25, 50, 80, 90, 100% and a second time in 100% ethanol. Each ethanol concentration was applied for 15 min. The ethanol solution was replaced with 100% acetone (anhydrous), repeated twice for 20 min. Infiltration of the embedding monomers was started by applying a 50% epoxy resin mixture in acetone anhydrous (Epoxy Embedding Medium kit, Sigma-Aldrich, 45359) for 1 h at room temperature, then changed to 100% epoxy resin without the accelerator DMP-30 for two hours at room temperature and finally, the solution was changed to fresh 100% epoxy resin with DMP-30. The samples were transferred to a 60 °C incubator and were left to polymerize for 72 h. After polymerization, the blocks were extracted from the cell culture plates by breaking the sides of the plate and peeling off the plastic bottom of the wells with a razor blade leaving only the resin and the attached glass coverslip. The coverslip was detached from the resin by dipping it briefly in liquid nitrogen. The blocks were trimmed with a razor blade and 70-nm ultrathin sections were obtained by cutting the blocks on a Reichert-Jung Ultracut E ultramicrotome using an Ultra 45° diamond knife (Diatome AG, Nidau, Switzerland). The sections were transferred onto formvar/carbon coated copper slot grids (S162-5, Plano EM, Wetzlar, Germany) for transmission electron microscopy. Samples were imaged with 120-kV FEI Tecnai G2 Spirit or 200-kV Tecnai F20 (ThermoFisher, Waltham, MA, US).

## Supplementary Information

Below is the link to the electronic supplementary material.

Supplementary Material 1

Supplementary Material 2

Supplementary Material 3

Supplementary Material 4

Supplementary Material 5

## Data Availability

The data supporting the findings of this study are available from the corresponding author upon reasonable request.
