# Supplementary material for: Sulfide silver autometallography to differentiate the ultrastructural localization of iron-carbohydrate complexes inside macrophages
Source: Sci Rep. 2025 Dec 8;15:43335. doi: 10.1038/s41598-025-27257-6 (PMC12686396; doi:10.1038/s41598-025-27257-6)
Supplement: Supplementary file 1 — Supplementary Material 1 [file 41598_2025_27257_MOESM1_ESM.docx]

***Supplementary information:***

**Sulfide silver autometallography to differentiate the** **ultrastructural localization of iron-carbohydrate complexes inside macrophages**

Anne-Marinette Cao^1^, Jules Duruz^1^, Mauro Sousa de Almeida^1^, Dimitri Vanhecke^1^, Amy E. Alston^2^, Reinaldo Digigow^2^, Beat Flühmann^2^, Alke Fink^1,3*^, and Barbara Rothen-Rutishauser^1^*

^1^Adolphe Merkle Institute, University of Fribourg, Chemin des Verdiers 4, 1700 Fribourg, Switzerland

^2^Vifor Pharma Group, Vifor Pharma Management Ltd

^3^Department of Chemistry, University of Fribourg, Chemin du Musée 9, Fribourg,

Switzerland

*Corresponding Authors

*Alke Petri-Fink email: [alke.fink@unifr.ch](mailto:alke.fink@unifr.ch) and *Barbara Rothen-Rutishauser email: [barbara.rothen@unifr.ch](mailto:barbara.rothen@unifr.ch)

***Supplementary figures***


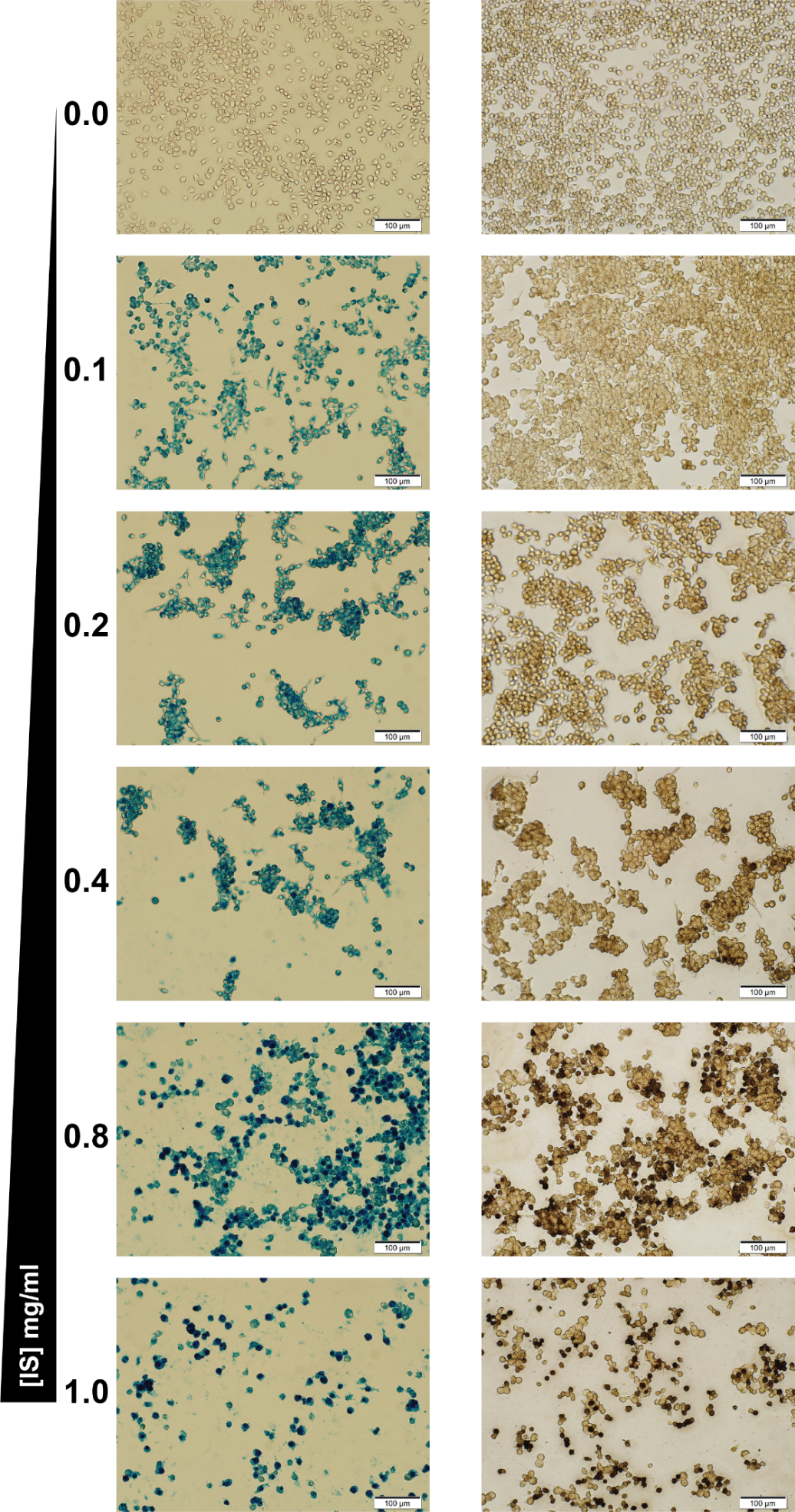


**Fig. S1. Macrophages treated with increasing doses of IS and imaged by Prussian blue (left) and ssAMG (right)**


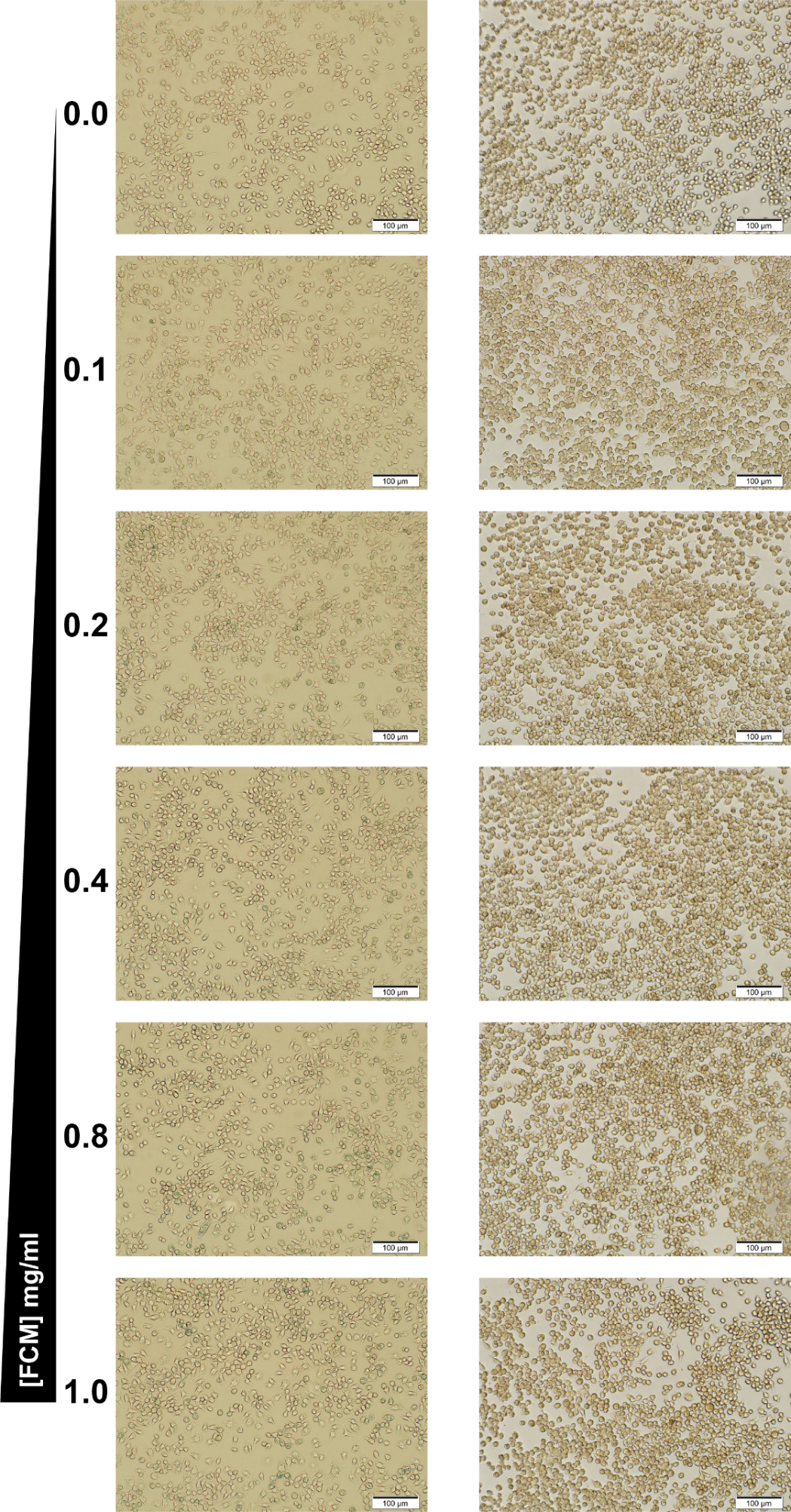


**Fig. S2. Macrophages treated with increasing doses of FCM and imaged by Prussian blue (left) and ssAMG (right)**


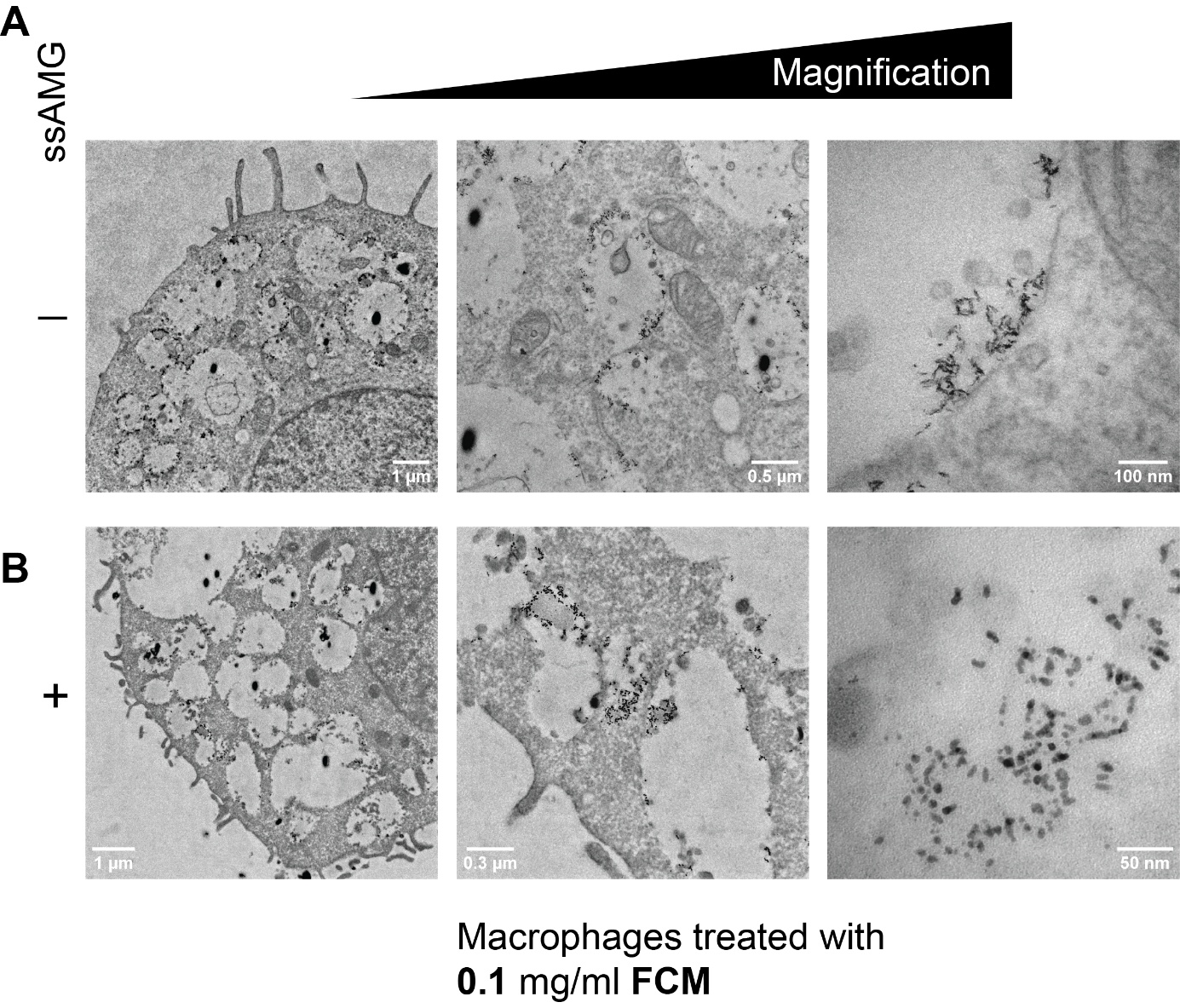


**Fig. S3. Representative TEM images of macrophages treated with 0.1 mg/ml FCM**


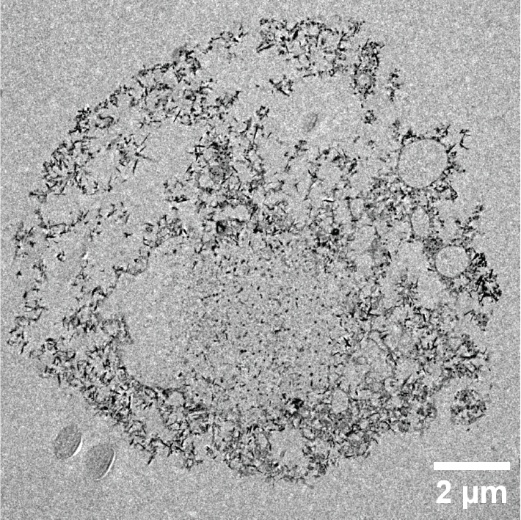


**Fig. S4. Representative TEM image of macrophages treated with 1 mg/ml IS**
