# Supplementary figures and images for: Sulfide silver autometallography to differentiate the ultrastructural localization of iron-carbohydrate complexes inside macrophages
Source: Sci Rep. 2025 Dec 8;15:43335. doi: 10.1038/s41598-025-27257-6 (PMC12686396; doi:10.1038/s41598-025-27257-6)

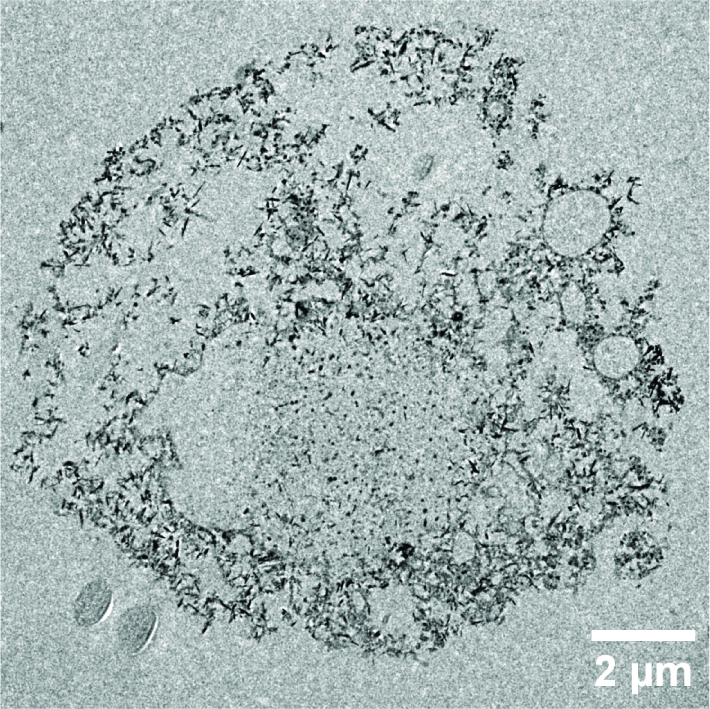

Supplement: Supplementary file 2 — Supplementary Material 2 [file 41598_2025_27257_MOESM2_ESM.tif]

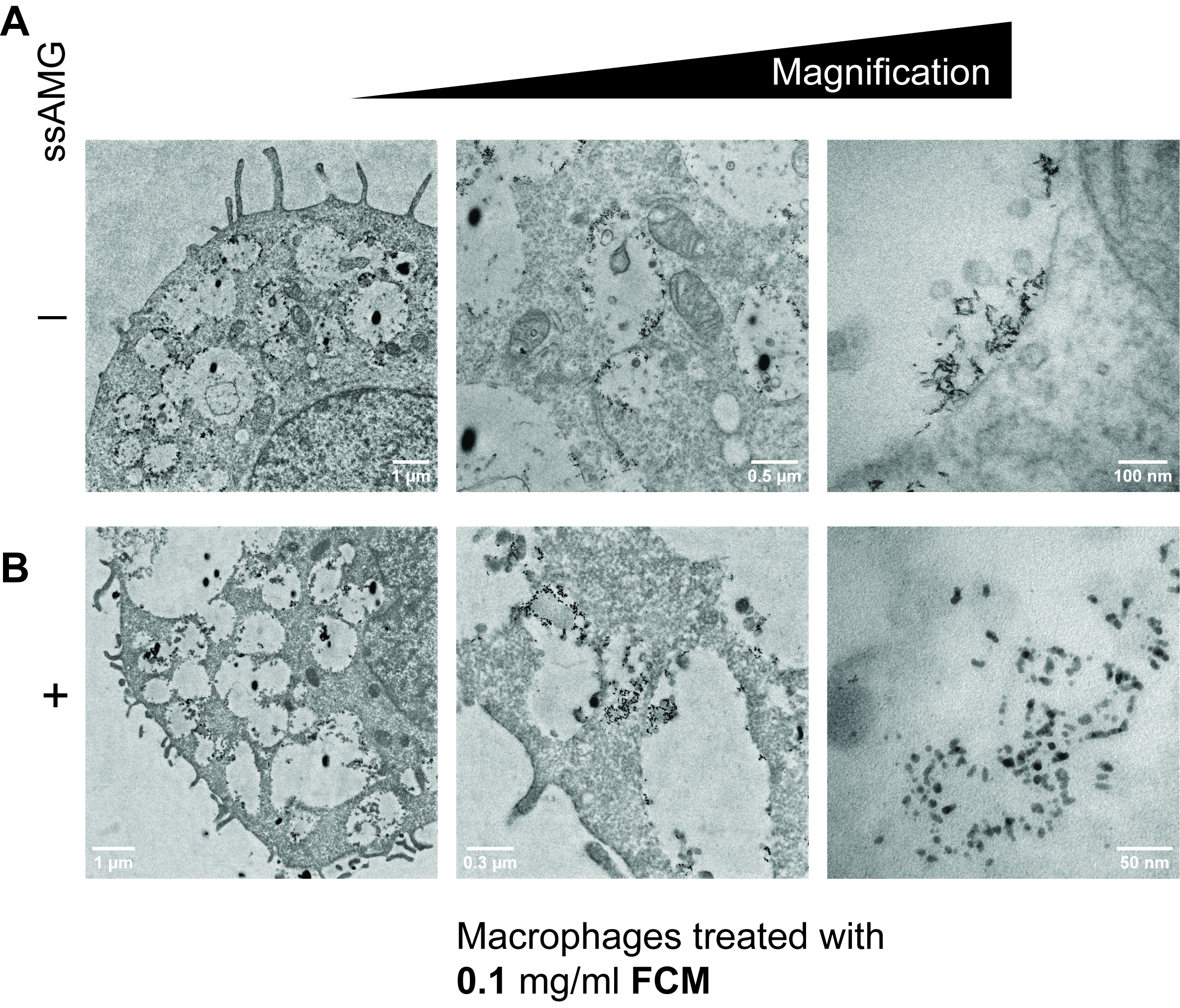

Supplement: Supplementary file 3 — Supplementary Material 3 [file 41598_2025_27257_MOESM3_ESM.tif]

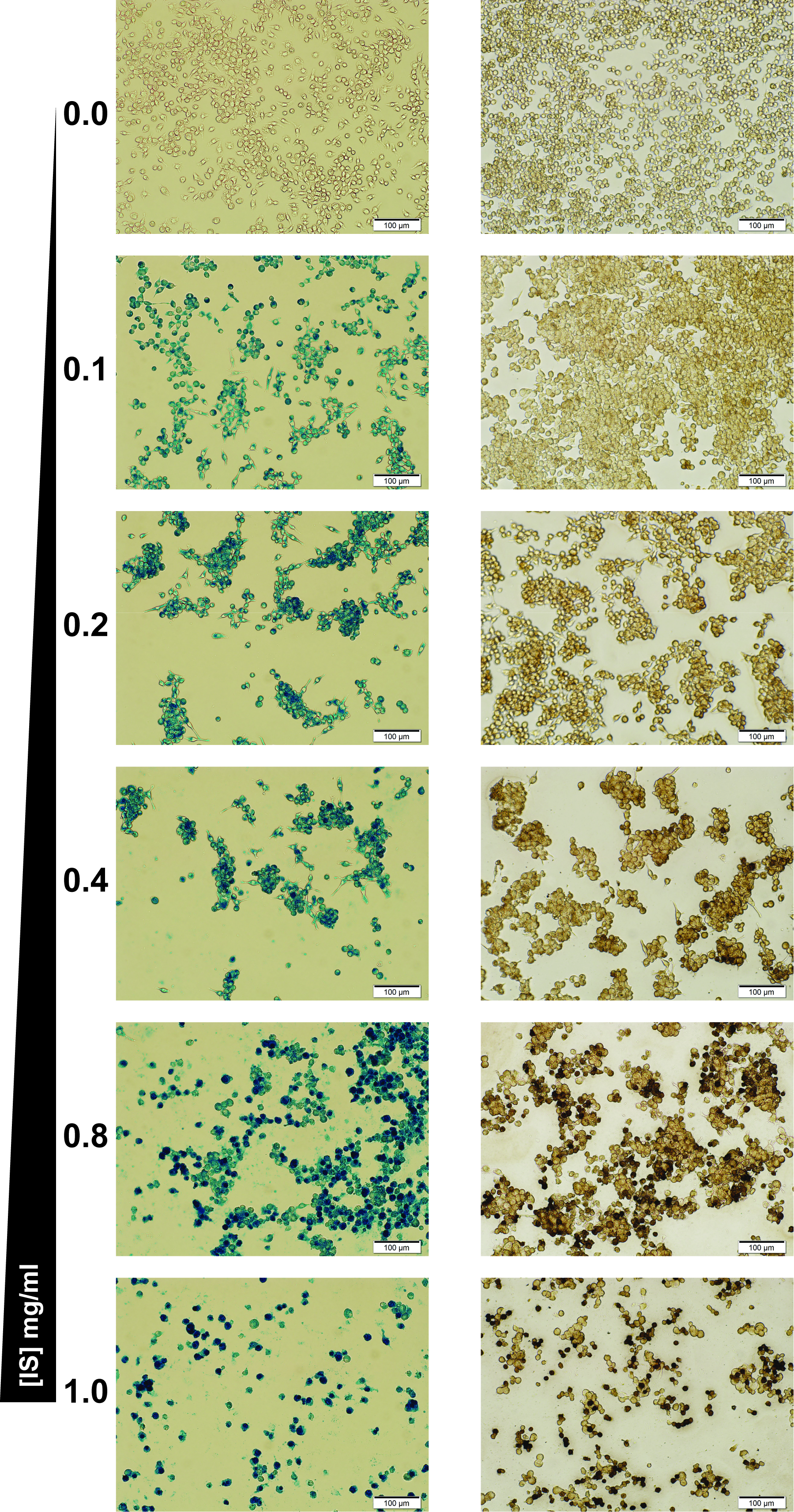

Supplement: Supplementary file 4 — Supplementary Material 4 [file 41598_2025_27257_MOESM4_ESM.tif]

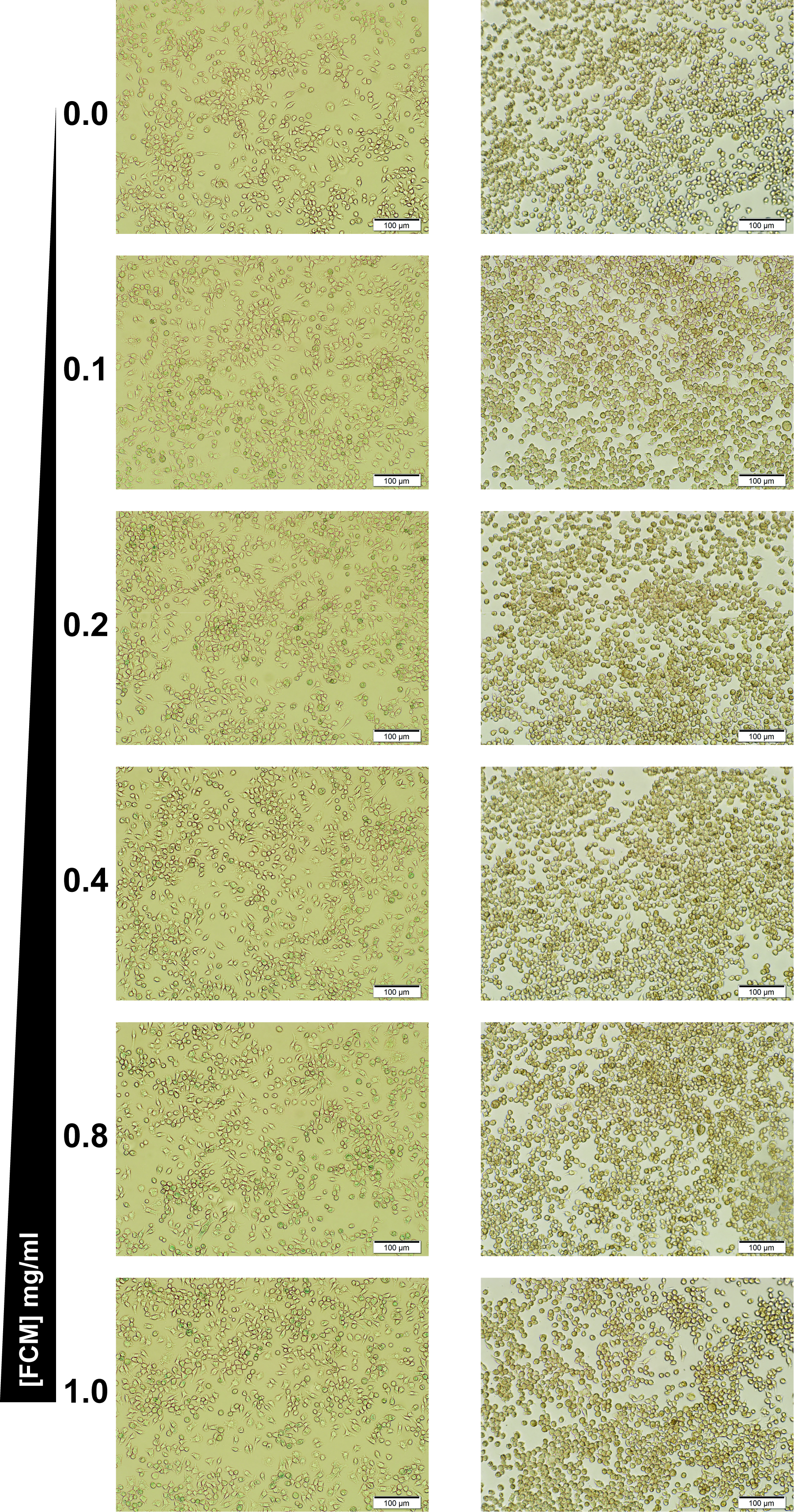

Supplement: Supplementary file 5 — Supplementary Material 5 [file 41598_2025_27257_MOESM5_ESM.tif]
